# Transgenerational inheritance of fetal alcohol effects on proopiomelanocortin gene expression and methylation, cortisol response to stress, and anxiety-like behaviors in offspring for three generations in rats: Evidence for male germline transmission

**DOI:** 10.1371/journal.pone.0263340

**Published:** 2022-02-10

**Authors:** Omkaram Gangisetty, Shaista Chaudhary, Ajay Palagani, Dipak K. Sarkar

**Affiliations:** Rutgers Endocrine Research Program, Department of Animal Sciences, Rutgers University, New Brunswick, New Jersey, United States of America; Massachusetts General Hospital, UNITED STATES

## Abstract

Previously it has been shown that fetal alcohol exposure increases the stress response partly due to lowering stress regulatory proopiomelanocortin (*Pomc*) gene expression in the hypothalamus via epigenetic mechanisms for multiple generations in mixed-breed rats. In this study we assess the induction of heritable epigenetic changes of *Pomc*-related variants by fetal alcohol exposure in isogenic Fischer 344 rats. Using transgenerational breeding models and fetal alcohol exposure procedures, we determined changes in hypothalamic *Pomc* gene expression and its methylation levels, plasma corticosterone hormone response to restraint stress, and anxiety-like behaviors using elevated plus maze tests in fetal alcohol-exposed offspring for multiple generations in isogenic Fischer rats. Fetal alcohol-exposed male and female rat offspring showed significant deficits in POMC neuronal functions with increased *Pomc* gene methylation and reduced expression. These changes in POMC neuronal functions were associated with increased plasma corticosterone response to restraint stress and increased anxiety-like behavior. These effects of fetal alcohol exposure persisted in the F1, F2, and F3 progeny of the male germline but not of the female germline. These data suggest that fetal alcohol exposure induces heritable changes in *Pomc*-related variants involving stress hyperresponsiveness and anxiety-like behaviors which perpetuate into subsequent generations through the male germline via epigenetic modifications.

## Introduction

The transgenerational effect of environmental toxicants was first demonstrated in rodent models in 2005 [1]. We are the first to demonstrate the transgenerational effects of alcohol in a rodent model of fetal alcohol exposure (FAE) [2]. Since then, transgenerational effects of alcohol and other drugs and stress during the developmental period have been demonstrated by many laboratories using rodent models [3–9]. Also, male-germline-mediated transgenerational transmission has been demonstrated for maternal diet effects through the F3 generation [10]. Transgenerational effects refer to phenomena that could not be ascribed to direct exposure of the target or stimulant on the affected organism. For instance, it can only affect the gestating embryo results in the altered phenotype in the second or third generation either through male or female transmission. However, the intergenerational effects occur when maternal exposure has direct effects on the altered phenotype of F1 and possibly on F2 offspring. Some human genetic studies provide evidence of intergenerational transmission of risk variants with the paternal lineage [11–13]. Additionally, sex-chromosome-wide association analysis suggested male-specific risk genes for alcohol dependence [14]. Transgenerational occurrence of reproductive diseases that have been linked to endocrine disruptors and endometriosis in women has also been documented [15]. Data of the Avon Longitudinal Study of Parents and Children (ALSPAC) indicate an association of grand maternal smoking with increased birth weight, birth length, and BMI in grandsons of non-smoking mothers but not in granddaughters—evidence for possible transgenerational transmission through the male germline in humans [16]. Epidemiological studies have shown an association of paternal alcohol consumption with deficiencies in offspring, such as decreases in birth weight and increases in ventricular septal defects in children [17], effects which are typically found with maternal alcohol exposure [18]. There is evidence that hyperactivity and diminished cognitive abilities in some children are related biologically to an alcoholic father [19, 20]. Alcohol abuse in humans is also shown to cause demethylation of normally hypermethylated imprinted regions in sperm DNA that is proposed to alter critical gene expression required for normal prenatal development resulting in offspring with features of fetal alcohol spectrum disorders (FASD) [21]. Our recent study using a human cohort of the Collaborative Initiative on Fetal Alcohol Spectrum Disorders (CIFASD) identified higher DNA methylation of the proopiomelanocortin (POMC) gene, which regulates stress axis functions, in mothers (and in their children) who gave birth to children with fetal alcohol spectrum disorder [22]. These data support the notion that epigenetic mechanisms might be involved in transgenerational effects of alcohol. Previously, by employing outbred Sprague-Dawley rats, we demonstrated that the fetal alcohol exposure increases methylation of the *Pomc* gene in association with stress hyperresponse in offspring for multiple generations [2]. Epigenetic states can be influenced by an underlying DNA sequence or be purely epigenetic (sequence-independent) [23]. An unbiased assessment of the heritability of epigenetic variation requires working with isogenic populations, in which any genetic contribution to variations can be neglected. Studies have reported various gene promoter DNA methylation changes following ethanol exposure, including Slc6a4 (serotonin transporter), Slc17a6 (glutamate transporter), oxidative stress, and peroxisome biogenesis genes. In addition, prenatal ethanol exposure led to decreased promoter methylation of polycomb repressive complex 1 members and TATA-box binding protein associated factors. A common endophenotype of fetal alcohol-exposed offspring is an elevated response of the hypothalamic pituitary adrenal axis. POMC is a critical regulator of the hypothalamic pituitary adrenal axis. We therefore assessed the induction of heritable epigenetic *Pomc*-related variants by fetal alcohol exposure in isogenic Fischer 344 (F344) rats maintained in standardized conditions in order to control both genetic and environmental sources of variations. The data of this study indicate a significant increase in methylation levels in the proximal part of *Pomc* promoter with the concomitant reduction in *Pomc* mRNA levels, in association with enhancement of stress hormone response to a stress challenge and increased anxiety-like behaviors in F1, F2, or F3 male progeny of the alcohol-fed mother’s male lineage.

## Materials and methods

### Animals

All rat studies were performed with approved protocol (PROTO0999900285) in compliance with the Association for the Assessment and Accreditation of Laboratory Animal Care and Rutgers University. Fischer rats were obtained from Harlan Laboratories (Indianapolis, IN) and were housed in a controlled condition at a constant temperature of 22°C and 12-hour light/dark cycles throughout the study. These rats were bred in our animal facility and used for this study. On gestational day (GD) 7 through 21, rats were fed with rat chow ad libitum (AD), a liquid diet containing ethanol (AF; 1.7–5.0% v/v from GD7-10 and 6.7% v/v from GD11-21; Bioserve Inc., Frenchtown, NJ), or pair-fed (PF; Bioserve) an isocaloric liquid control diet (with alcohol calories replaced by maltose-dextrin). Previous studies have shown that the peak blood ethanol concentration is achieved in the range of 120–150 mg/dl in pregnant dams fed with this ethanol-containing liquid diet [24]. AF and PF litters were cross-fostered, and the litter size was maintained at 8 pups/dam. Only one pup from each litter was used in an experimental measure. Transgenerational studies were conducted by breeding AF, PF, or AD rats with control animals of the opposite gender to produce two germlines. We generated the male germline (AFM or PFM) by breeding male (AF or PF) rats and their male offspring with control (AD) female rats and the female germline (AFF or PFF) by breeding female (AF or PF) rats and their female offspring with control (AD) male rats. All rats were sacrificed by rapid decapitation to avoid stress at 60–90 days after birth, and tissues were collected for further experimentation.

### Corticosterone response to restraint stress

Corticosterone response to restraint stress at various time points were determined in male and female rat offspring (on day of diestrus) at day 60 after birth from male and female germlines. Animals were restrained by placing them in a transparent plexiglass restrainer for 1 hour (between 12:00 and 14:00h). Blood samples were collected at various time points before (0h) and after restraint. Female rats were restrained on the day of diestrus to avoid the influence of the fluctuating levels of steroid on the corticosterone response. Plasma corticosterone levels were measured using corticosterone ELISA kit (IBL America, Mineopolis, MN) following manufacturer’s instructions.

### Elevated plus maze

The elevated plus maze (EPM) test was conducted to assess anxiety-related behaviors as described previously [25]. The apparatus with a shape of a ‘‘+”consisted of two open arms and two closed arms. The arms extended from a central platform, 10 × 10 cm^2^ in area, and the maze was elevated to a height of 55 cm from the floor. Rats placed on the junction of the open and closed arms were allowed to freely explore the maze for 300 second. The time spent in the open and closed arms was recorded using ANY-Maze video tracking software. Proper care was taken to avoid any sudden noise or disturbance. The apparatus was cleaned with 75% ethanol after each trial.

### Gene expression analysis by qRT-PCR

*Pomc* gene levels in the mediobasal hypothalamus (MBH) samples were measured by quantitative real-time polymerase chain reaction (RT-PCR) (SYBR Green assay) as described previously [26]. Total RNA was isolated from tissue samples using an RNeasy kit (Qiagen, Valencia, CA). Total RNA (1 μg) was converted to first-strand complementary DNA (cDNA) using a high-capacity cDNA reverse transcription kit (Life Technologies). The primer sequences used for *Pomc*, *Gapdh*, *18S*, and *Rpl19* are presented in Table 1. Real-time quantitative PCR was performed at 95°C for 5 minutes, followed by 40 cycles of 95°C for 15 seconds, 60°C for 30 seconds, and 72°C for 40 seconds in the Applied Biosystems 7500 real-time PCR system (Foster City, CA). The quantity of target genes (*Pomc*) and the three reference genes (*Gapdh*, *18S*, and *Rpl19*) were measured using the standard curve method. Target-gene expression was normalized with reference gene expression levels.

**Table 1:** Primer sequences.

Primer Name	Sequence
*Pomc* FP	5’-CAAGAGGGAGCTGGAAGGCGAGC-3’
*Pomc* RP	5’-TCACTGGCCCTTCTTGTG-3’
*Gapdh* FP	5’-AGACAGCCGCATCTTCTTGT-3’
*Gapdh* RP	5’-CTTGCCGTGGGTAGAGTCAT-3’
*18s* FP	5’ GTAACCCGTTGAACCCCATT 3’
*18s* RP	5’ CCATCCAATCGGTAGTAGCG 3’
*rpl-19* FP	5’ AATCGCCAATGCCAACTCTCG 3’
*rpl-19* RP	5’ TGCTCCATGAGAATCCGCTTG 3’
*Pomc* BS FP	5’ AATGTTAGGAAGGTAGATG-3’
*Pomc* BS RP	5’-Biotin-TCCCTATCACTCTTCTCTCTTCTT-3’
*Pomc* seq FP	5’-ATTAAGTTTTTTTTGATTAT-3’

Forward primer (FP), reverse primer (RP), Bisulfite sequencing forward primer (BS FP), Bisulfite sequencing reverse primer (BS RP), Sequencing forward primer (seq FP).

### DNA methylation analysis by bisulfite pyrosequencing

In order to characterize the extent of cytosine methylation of CpG dinucleotides in the *Pomc* proximal promoter, we designed bisulfite sequencing primers in the CpG island. The methods for *Pomc* bisulfite pyrosequencing were as described previously [27]. A MethPrimer program was used to assess the CpG island and bisulfite sequencing primers for *Pomc* gene promoter (Table 1). Genomic DNA was extracted from MBH samples using DNeasy kit (Qiagen). About 1 ug of DNA was subjected to bisulfite treatment using EZ DNA methylation kit (Zymo Research, Orange, CA). Following bisulfite treatment, PCR reaction was performed with bisulfite sequencing primers using Pyromark PCR reagents (Qiagen, Valencia, CA) as detailed in the manufacturer’s protocols. Pyrosequencing was carried out using a sequencing primer on a PSQ-HS-96A model pyrosequencer (Qiagen). In the study, we analyzed one control C in a non-CpG background for efficient C→T conversion. This CpG site provides a built-in control for bisulfite treatment. Each CpG in the study was analyzed by comparing C/T peaks. The peak heights in the resulting pyrogram report the ratio of cytosine to thymine at each analyzed CpG site and reflects the portion of methylated DNA. The percentage of methylation was calculated and represented using the following formula:

% of methylation = percent C remaining as C in each target CpGX Control C → T %.

It is a highly sensitive method to detect and quantify small changes in methylation levels. The built-in control ensures the reliability of the results.

### Statistical analysis

The data shown in the figures and text are mean ± SEM. Comparisons between groups were made using one-way analysis of variance (ANOVA) with post hoc analysis using the Newman Keuls posttest. Two-way ANOVA with Bonferroni post hoc tests were used for multiple comparisons. Significance was set at p<0.05. We used Prism 5.0 software for the analysis.

## Results

### Fetal alcohol exposure reduces *Pomc* gene expression in the mediobasal hypothalamus of male offspring rats for multiple generations

**Fig 1:**
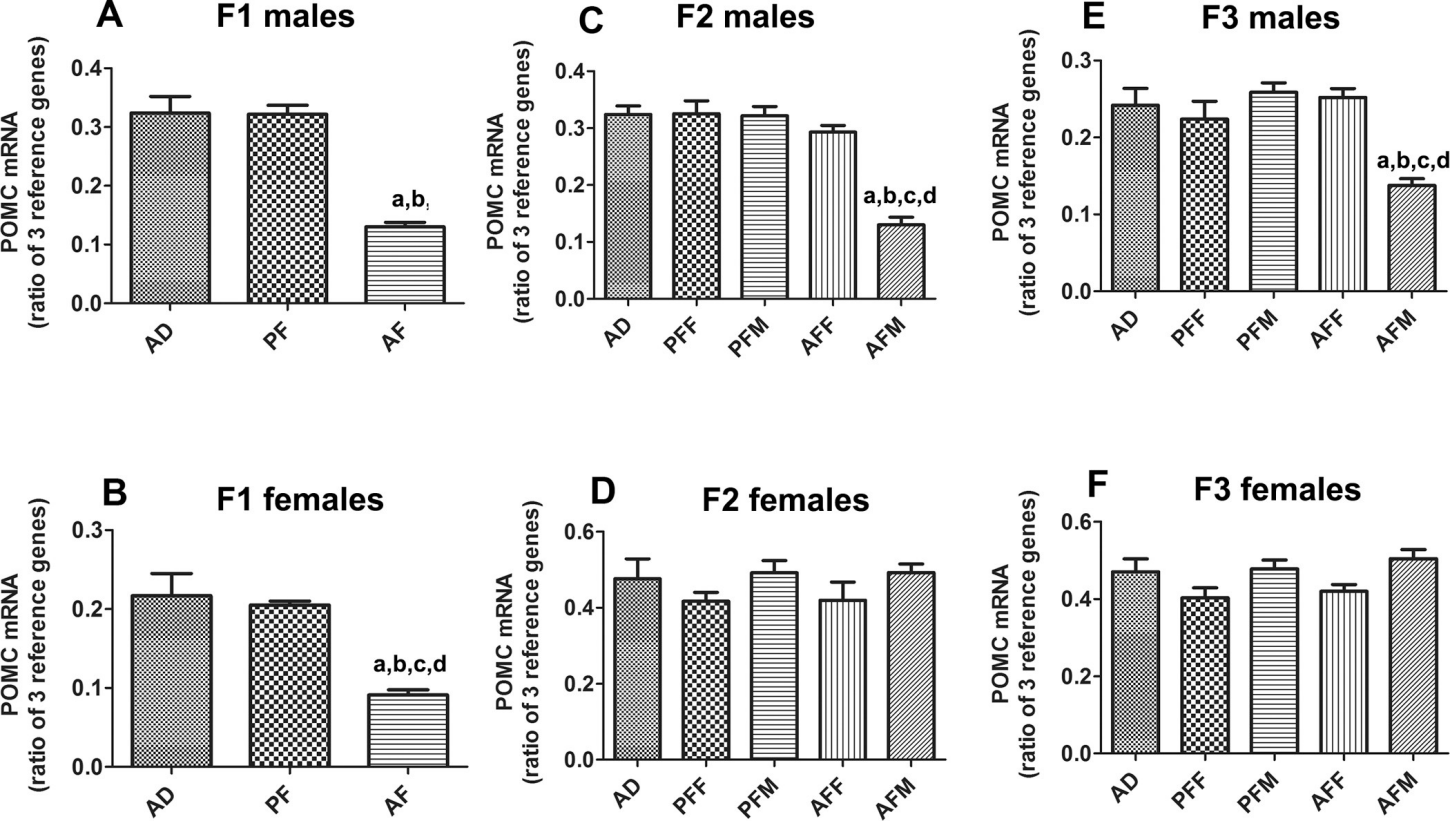
Transgenerational changes in *Pomc* mRNA levels in fetal alcohol exposed offspring for three generations. Changes in the level of *Pomc* mRNA in the mediobasal hypothalamus of male and female offspring of pregnant rats fed an alcohol-containing liquid diet (AF), pair-fed an isocaloric liquid diet (PF), or ad lib-fed rat chow (AD) in the F1 generation (A, B), and in male and female offspring bred from the alcohol-fed male germline (AFM) or female germline (AFF) or pair-fed male germline (PFM) or female germline (PFF) in F2 (C, D) and F3 (E, F) generations. Data are mean ± SEM (n = 8 rats) and analyzed using one-way ANOVA followed by the Student Newman-Keuls post hoc test, and significant differences are indicated by ^a^*p*<0.05, compared to AD; ^b^*p*<0.05, compared to PF or PFF; ^c^*p*<0.05, compared to PFM; ^d^*p*<0.05, compared to the AFF group.

To determine the impact of fetal alcohol exposure in the inheritance of stress axis abnormalities, we first measured the changes in gene expression of a stress regulatory hormone *Pomc* in offspring during three generations. In the transgenerational study, we employed selected breeding procedures to determine if the transgenerational transmission of FAE effects is male or female germline specific. Determination of *Pomc* mRNA levels in the mediobasal hypothalamus (MBH) revealed that feeding an alcohol-containing liquid diet (AF), but not pair-feeding an isocaloric liquid diet (PF), significantly reduced the levels of *Pomc* gene expression in the MBH of F1 generation male and female offspring (Fig 1A and 1B). The suppressive effect of fetal alcohol on *Pomc* gene expression persisted in subsequent F2 and F3 generation offspring in a sex- and germline-dependent manner. We found the *Pomc* gene expression level was significantly lower in male offspring derived from the fetal alcohol-fed male germline (AFM) in the F2 and F3 generations (Fig 1C and 1E). However, the *Pomc* gene expression level did not change in male offspring derived from the fetal alcohol-fed female germline (AFF) or in female offspring derived from the alcohol-fed male germline (AFM) or female germline (AFF) in the F2 and F3 generations (Fig 1D and 1F). We also did not find any effect of pair-feeding the isocaloric liquid diet (PF) on *Pomc* gene expression in male or female offspring of any germline during F2 to F3 generation breeding (Fig 1). These data are in agreement with the previous finding that fetal alcohol exposure -induced changes in *Pomc* gene expression is transmitted to multiple generations via the male germline in Sprague Dawley rats [2].

### Fetal alcohol exposure induces transgenerational effect on *Pomc* promoter DNA methylation in the mediobasal hypothalamus of male offspring rats for multiple generations

**Fig 2:**
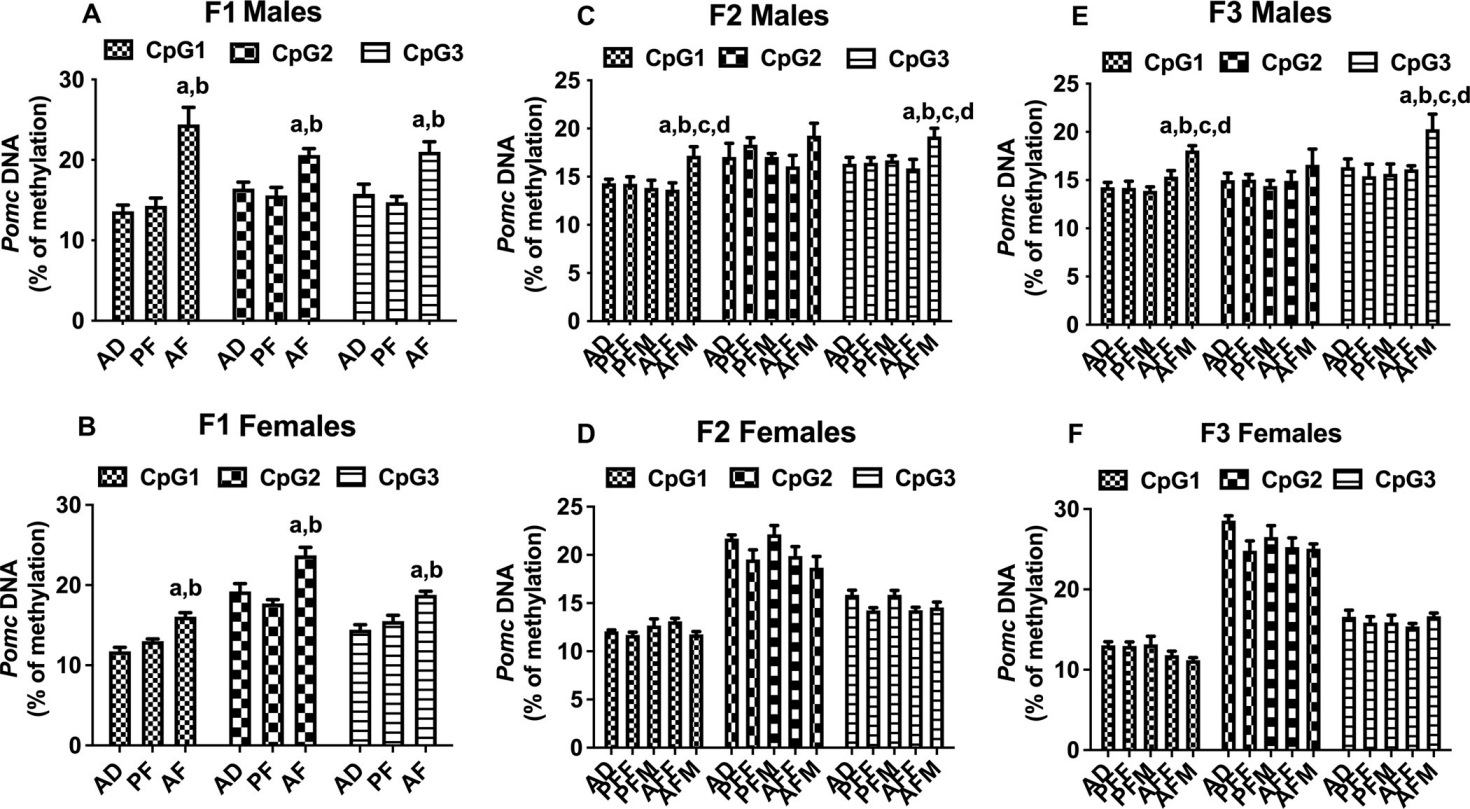
Transgenerational changes in *Pomc* DNA methylation levels in fetal alcohol exposed offspring for three generations. Transgenerational changes in *Pomc* DNA promoter methylation in male and female AF, PF, or AD offspring in the F1 generation (A, B), and in AFM, AFF, PFM, and PFF in F2 (C, D) and F3 (E, F) generations. *Pomc* gene methylation at various CpG sites of *Pomc* promoter CpG islands CpG1 (-238), CpG2 (-224), and CpG3 (-216) were measured using pyrosequencing. Data are mean ± SEM (n = 9 rats) and analyzed using one-way ANOVA followed by the Student Newman-Keuls post hoc test. Significant differences are indicated by ^a^*p*<0.05, compared to AD; ^b^*p*<0.05, compared to PF or PFF; ^c^*p*<0.05, compared to PFM; ^d^*p*<0.05, compared to the AFF group.

It is now well recognized that epigenetic modifications of a gene play an important role in the control of its transcription. Global or site-specific methylation of CpG sites near and within the regulatory regions of genes is often associated with transcriptional inactivity and gene suppression [28]. Therefore, we determined if the fetal alcohol exposure -induced changes in *Pomc* gene expression is associated with the altered promoter DNA methylation. Using the Methyl Primer Express v1.0 program (ABI, Waltham, MA), we previously identified three potential CpGs: CpG1 (-238), CpG2 (-224), and CpG3 (-216) in *Pomc* promoter CpG islands. We also showed FAE altered the methylation status of these CpGs by employing pyrosequencing, which is highly sensitive in determining methylation status at single CpG resolution [27]. Using the pyrosequencing methods, we show here FAE alters *Pomc* gene methylation at various CpG sites of *Pomc* promoter CpG islands in a sex- and germline-dependent manner (Fig 2). FAE increased methylation levels of all three different CpGs across *Pomc* promoter CpG islands in the MBH of both male and female F1 offspring (Fig 2A and 2B). In F2 and F3 generation males, *Pomc* promoter methylation levels of CpG1 (-238) and CpG3 (-216) were significantly increased only in offspring of the male germline (AFM) but not in the female germline (AFF) (Fig 2C and 2E). In F2 and F3 generation females, *Pomc* promoter methylation levels did not change in offspring of AFM and AFF germlines (Fig 2D and 2F). This method of bisulfite pyrosequencing is a highly sensitive one to detect even small changes in DNA methylation of each CpG. We previously measured changes in DNA methylation of these three potential CpGs of POMC promoter and its correlation with expression (2, 26). Although the increased changes in DNA methylation of these CpGs are small, they are correlated with reduced expression of POMC as we reported previously. These data are in agreement with our previous findings that fetal alcohol exposure increased *Pomc* gene methylation is transmitted to multiple generations via the male germline in outbred Sprague Dawley rats [2]. Therefore, the male-germline-specific transgenerational effects of FAE on *Pomc* gene transcription is related to epigenetic modifications of the gene promoter and not to genetic variations of the animals.

### Transgenerational effects of fetal alcohol exposure on corticosterone response to a restraint stress challenge

**Fig 3:**
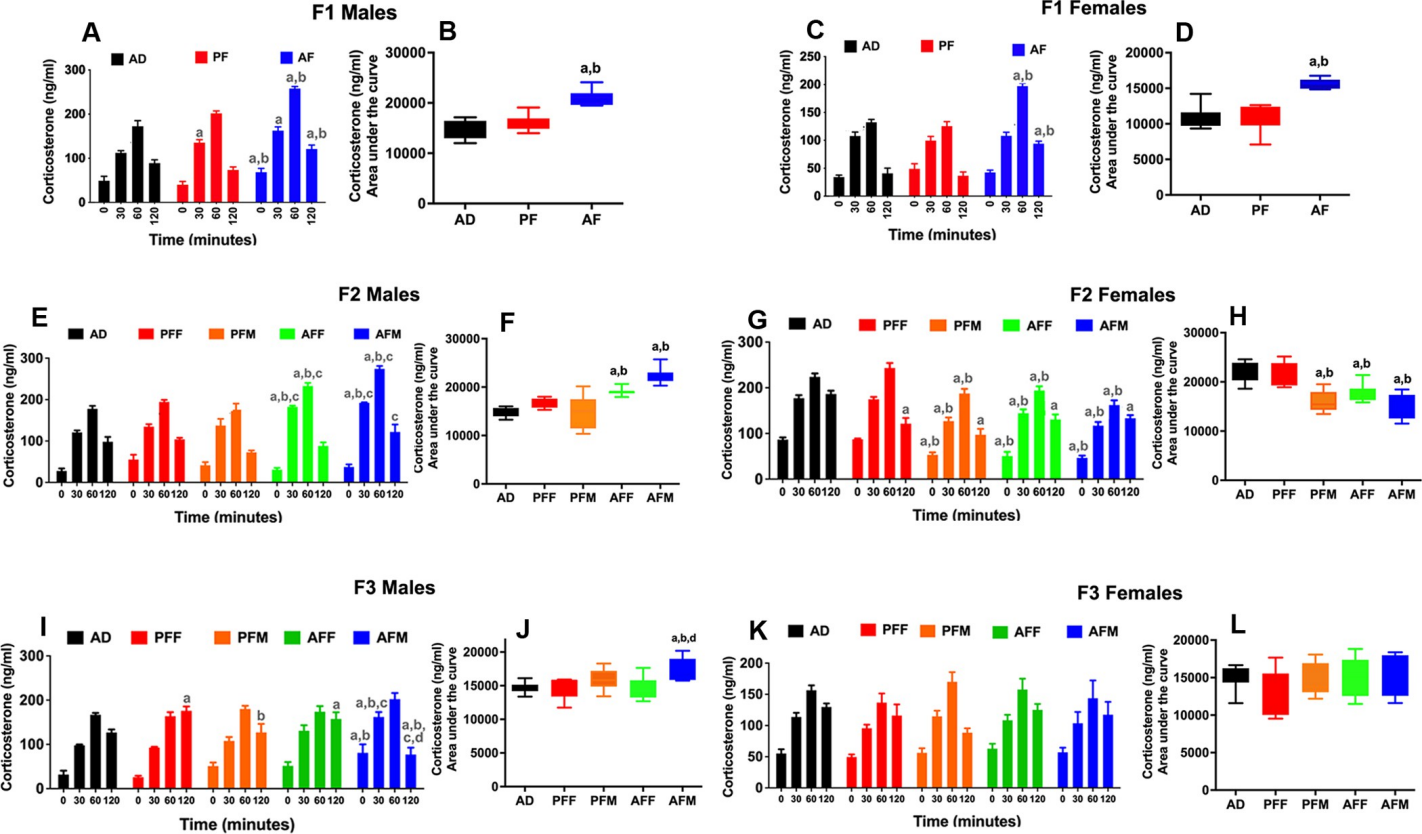
Transgenerational changes in plasma corticosterone response to restraint stress in fetal alcohol exposed offspring. Corticosterone responses to restraint stress were evaluated in male and female rat offspring (on day of diestrus) at day 60 after birth from male and female e germlines. Time-dependent changes of cortisol before, during, and after restraint are shown for each group for F1 males and females (A, C), F2 males and females (E, G), and F3 males and females (I, K). Data are mean ± SEM (n = 8 rats) and analyzed using two-way ANOVA followed by Tukey’s multiple comparison post hoc test. Significant differences of the hormone response at the same time point between groups are indicated by ^a^*p*<0.05, compared to AD; ^b^*p*<0.05, compared to PF or PFF; ^c^*p*<0.05, compared to PFM; ^d^*p*<0.05, and compared to the AFF group. Total levels of corticosterone released during the restraint stress study as determined by the area under curve for F1 (B, D), F2 (F, H), and F3 (J, L) male and female offspring from different germlines (n = 8 rats). Data are mean ± SEM (n = 8 rats) and analyzed using one-way ANOVA followed by the Student Newman-Keuls post hoc test. ^a^*p*<0.05, compared to AD; ^b^*p*<0.05, compared to PF or PFF; ^d^*p*<0.05, and compared to the AFF group.

To evaluate whether the transgenerational effect of fetal alcohol exposure applies to some endophenotypes of the *Pomc* defect, we determined stress hormone response to restraint stress in F1–F3 rat offspring. It was observed that fetal alcohol exposure increased plasma levels of corticosterone in response to 60 minutes of restraint stress, which persisted 60 minutes following restraint in both male and female offspring of F1 progeny (Fig 3A and 3C). The total amount of corticosterone released during the 120-minute period, as determined by the area under the curve, was also increased in both sexes in this generation’s progeny (Fig 3B and 3D). In the F2 male progeny, the corticosterone response to restraint stress was significantly elevated in both AFM and AFF germlines as determined by time response (Fig 3E and 3G) and the area under the curve (Fig 3F). In the F2 female progeny, the corticosterone response to stress fluctuated and showed a moderate reduction in the control-fed male germline (PFM), as well as in AFF and AFM groups (Fig 3G and 3H). In the F3 male progeny, the corticosterone response to restraint stress was significantly elevated in AFM but not in the AFF germline as determined by time response (Fig 3I) and the area under the curve (Fig 3J). No changes of corticosterone response to restraint were found in F3 female progeny in AFF or AFM germlines (Fig 3K and 3L). These results suggest that fetal alcohol’s effects on stress axis abnormalities perpetuate into subsequent generations through the male germline.

### Transgenerational effects of fetal alcohol exposure on anxiety-like behaviors

**Fig 4:**
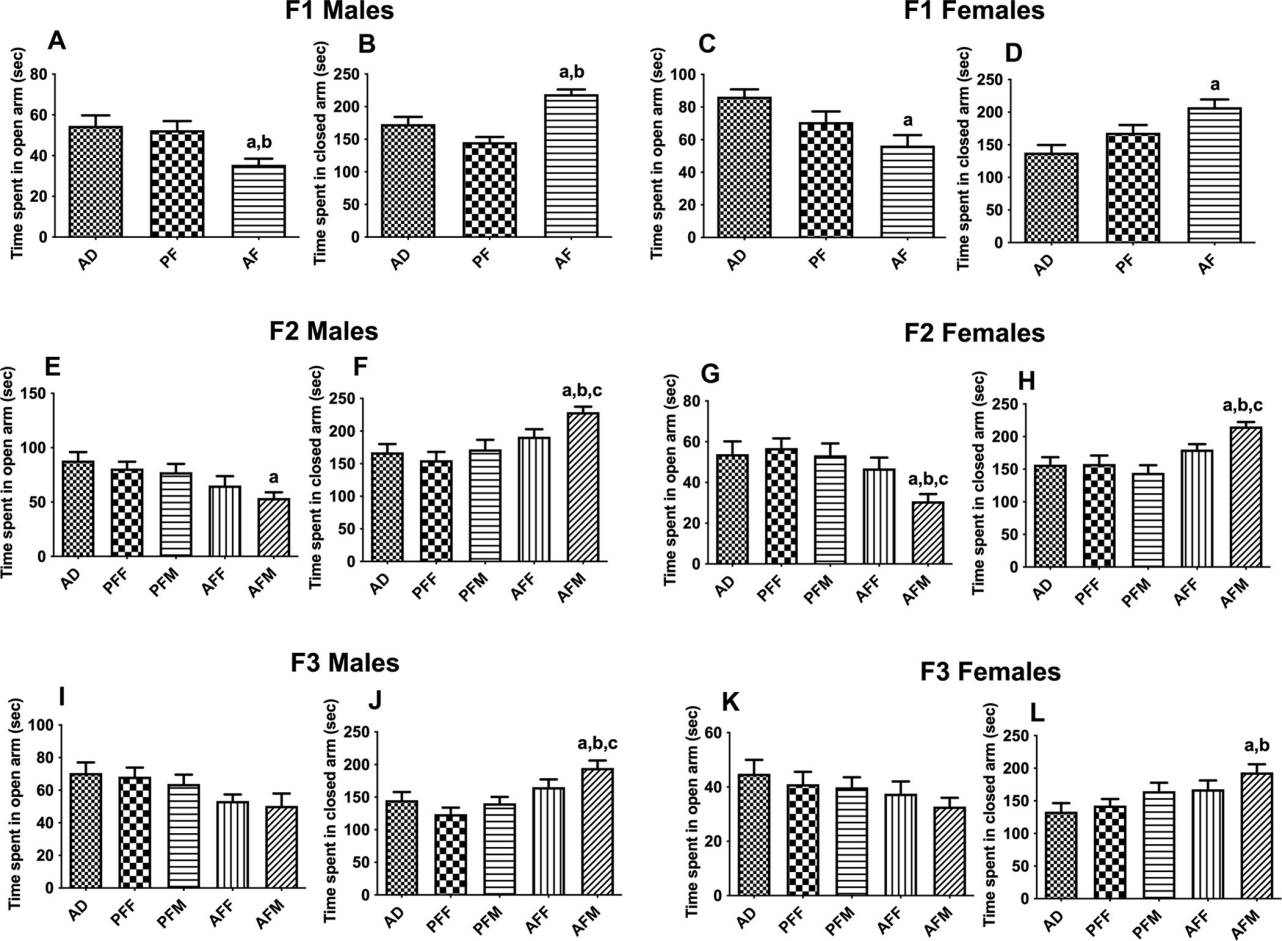
Transgenerational changes on anxiety-like behaviors in fetal alcohol exposed offspring. Effect of prenatal ethanol exposure on anxiety-like behaviors as determined by the elevated plus maze (EPM) in male and female offspring of F1, F2, and F3 generations. For EPM analysis, time (sec) in closed and open arms was analyzed in F1-F3 generation rats. Data are expressed as mean ± SEM, n = 8 in each condition. The data were analyzed using one-way ANOVA followed by the Student Newman-Keuls post hoc test and significant differences are indicated by ^a^*p*<0.05, compared to AD; ^b^*p*<0.05, compared to PF or PFF; ^c^*p*<0.05, compared to PFM; ^d^*p*<0.05, compared to the AFF group.

We also tested the effects of fetal alcohol exposure on anxiety-like behavior. The Elevated Plus Maze data showed that fetal alcohol exposure induced a significant increase in the amount of time spent in closed arms and a significant decrease in time spent in open arms in the AF group when compared to AD and PF groups in both the male and female F1 offspring (Fig 4A–4D). In the F2 generation, the male offspring of the male germline (AFM) also showed a significant increase in the amount of time spent in closed arms and a significant decrease in time spent in open arms as compared to controls, whereas the female germline (AFF) did not show any significant change in time spent in closed and open arms as compared to controls in both male and female rats (Fig 4E–4H). In the F3 generation, the male and female progeny of the male germline (AFM) also showed a significant increase in the amount of time spent in closed arms as compared to controls (Fig 4I–4L). However, the F3 male and female progeny of the female germline (AFF) did not show any change. Also, no significant changes were observed in the amount of time spent in open arms in F3 generation male and female progeny (Fig 4I–4L).

## Discussion

Our previous studies in outbred Sprague Dawley rats suggest that the fetal alcohol exposure -induced methylation state of the *Pomc* gene and its endophenotypes is inherited through the male germline. Epigenetic states can be influenced by an underlying DNA sequence or be purely epigenetic (sequence-independent) [23]. We therefore assess the induction of heritable epigenetic *Pomc*-related variants by fetal alcohol exposure in isogenic F344 rats maintained in standardized conditions in order to control both genetic and environmental sources of variations. The data of this study indicate that in F344 rats, the F1, F2, and F3 male progeny of the AF mother’s male lineage had a significant increase in methylation levels in the proximal part of the *Pomc* promoter with the concomitant reduction in the *Pomc* mRNA level. DNA methylation or expression levels of *Pomc* did not change in F1, F2, or F3 male progeny of the PF mother’s male lineage as compared to controls. These data provide support to the view that FAE-induced epigenetic marks on the *Pomc* gene are heritable.

We showed here that fetal alcohol exposure induces long-lasting hypermethylation of the *Pomc* gene, since the percentage of the cytosine methylation of CpG sites adjacent to the gene transcription start site for *Pomc* was higher in FAE animals than in controls. Methylation of the proximal region of the promoter is known to correlate closely with a negative effect on gene expression [24]. Moreover, deletions or mutations in these promoter regions cause a decrease in gene expression for *Pomc* [29, 30]. Hence, increased DNA methylation in the key epigenetic island in the promoter regions of the *Pomc* gene could have reduced expression levels of this gene in the hypothalamus, which has been demonstrated in the fetal alcohol exposed offspring. In this context, it should be emphasized that administration of 5-azadeoxycytidine (AZA), a blocker of DNA methylation, DNA methyltransferases, prevents FAE-induced alteration of POMC gene expression and further transmission to the next generation via the offspring [2]. Overall, these findings provide evidence that FAE effects on *Pomc* hypermethylation and stress axis abnormalities persisted throughout adulthood and perpetuated into subsequent generations through the male germline [2].

The transgenerational effect of fetal alcohol exposure was also noted in some endophenotypes of the *Pomc* defect. We found an increase in the restraint stress-induced increase in corticosterone levels in male offspring of the F1, F2, and F3 progeny of the male germline. These male germline-dependent corticosterone responses to stress were like those of POMC gene methylation changes in F1 to F3 FAE offspring. Additionally, previous studies have shown that POMC neuronal transplants prevent a FAE-induced increase in corticosterone response to stress [31]. Therefore, FAE-induced epigenetic modification of the *Pomc* gene may also contribute, at least partly, to the multigenerational defects of the stress response in FAE offspring.

Behavioral testing across three generations also indicated that fetal alcohol exposure generates stable transgenerational increases in anxiety-like behaviors, persisting via the male germline to unexposed offspring. In our study, fetal alcohol exposure increased anxiety-like behaviors in the elevated plus maze because the FAE offspring showed an increased amount of time spent in the closed arm. These behavioral changes persisted in only male germline-derived male and female offspring for three generations. We also found a significant decrease in the amount of time spent in the open arms in both male and female offspring of the male germline for two generations and a moderate decrease (did not reach significant differences) in the third generation male and female progeny of the male germline. Previously it has been shown that transplantation of POMC neurons in the FAE offspring increased the number of entries into the open arms and reduced the time spent in closed arms relative to the control animals in an elevated plus maze [32], suggesting that POMC neurons contribute to the control of the anxiety-like behaviors in response to novel environments in fetal alcohol exposed animals. Thus, fetal alcohol exposure -induced POMC deficiency contributes at least partly to the increased anxiety-like behaviors in FAE offspring of the male germline for multiple generations. It should be noted that the transgenerational effect of fetal alcohol exposure on anxiety-like behavior is expressed in both male and female offspring of the male germline, while the transgenerational effect of fetal alcohol exposure on POMC gene methylation and expression are observed only in the male progeny of the male germline. Hence, there may be other undetermined factors which also contribute to the changes of these behaviors in female FAE offspring for male germline-dependent transgenerational transmission.

Epigenetic transgenerational inheritance was reported in a number of animal studies using exposure to environmental toxicants, diet restrictions, hormonal imbalance, and stress factors. Environmental exposure to vinclozoline, an endocrine disruptor, increases stress and anxiety in the F3 generation [33]. A maternal high-fat diet increases susceptibility of male offspring to liver disease through epigenetic reprogramming of lipid metabolism and inflammatory responses [34]. Postnatal trauma elicits depressive-like behavior for up to three generations [35]. A study has provided evidence for the implication of sperm microRNAs in transgenerational inheritance of trauma-induced phenotypes across generations [36]. Fetal alcohol exposure elicits epigenetic marks on interferon-γ that pass through multiple generations via the male germ line, providing evidence of FAE’s adverse effect on the immune system [37]. Some human studies provided evidence for epigenetic transgenerational inheritance. Transgenerational transmission of trauma has been studied in the offspring of veterans and refugee families [38, 39]. Studies have shown that a grandparent with a major depressive disorder with alcohol abuse increased the risk of an individual having major depressive behavior [40].

In conclusion, our data suggest that FAE induces heritable changes in POMC gene expression and affects the stress response and anxiety-like behaviors via epigenetic modifications for multiple generations via the male germline.

## Supporting information

S1 Table Power analysis of the POMC expression and methylation data to calculate sample size. (DOCX)
