# Supplementary material for: Transgenerational inheritance of fetal alcohol effects on proopiomelanocortin gene expression and methylation, cortisol response to stress, and anxiety-like behaviors in offspring for three generations in rats: Evidence for male germline transmission
Source: PLoS One. 2022 Feb 10;17(2):e0263340. doi: 10.1371/journal.pone.0263340 (PMC8830645; doi:10.1371/journal.pone.0263340)
Supplement: S1 Table — (DOCX) [file pone.0263340.s001.docx]

S1 Table

Power analysis of the POMC expression and methylation data to calculate sample size

| Figure | Experiment  description | Treatment group (mean) | Treatment comparison  (Standard deviation) | Calculated sample size for specified power |
| --- | --- | --- | --- | --- |
| 1A | F1 male | AD (0.324)  PF (0.322)  AF (0.130) | AD vs AF (0.1369)  PF vs AF (0.1354) | N=8 |
| 1B | F1 Female | AD (0.217)  PF (0.205)  AF (0.092) | AD vs AF (0.089)  PF vs AF (0.081) | N=8 |
| 1C | F2 male | AD (0.324)  PFF (0.326)  PFM (0.322)  AFF (0.293)  AFM (0.130) | AD vs AFM (0.1374)  PFF vs AFM (0.1383)  PFM vs AFM (0.1357)  AFF vs AFM (0.1154) | N=8 |
| 1E | F3 male | AD (0.242)  PFF (0.224)  PFM (0.259)  AFF (0.252)  AFM (0.138) | AD vs AFM (0.0737)  PFF vs AFM (0.0609)  PFM vs AFM (0.0857)  AFF vs AFM (0.0809) | N=8 |
| 3A | F1 male (CpG1)  F1 male (CpG2)  F1 male (CpG3) | AD (13.62)  PF (14.28)  AF (24.86)  AD (16.3)  PF (15.97)  AF (20.62)  AD (15.8)  PF (14.12)  AF (21) | AD vs AF (7.948)  PF vs AF (7.481)  AD vs AF (3.055)  PF vs AF (3.288)  AD vs AF (3.677)  PF vs AF (4.865) | N=8  N=8  N=8 |
| 3B | F1 female (CpG1)  F1 female (CpG2)  F1 female (CpG3) | AD (11.54)  PF (13.13)  AF (16.05)  AD (19.2)  PF (17.73)  AF (23.72)  AD (15.03)  PF (15.86)  AF (18.44) | AD vs AF (3.189)  PF vs AF (2.065)  AD vs AF (3.196)  PF vs AF (4.236)  AD vs AF (2.411)  PF vs AF (1.824) | N=8  N=8  N=8 |
| 3C | F2 male (CpG1)  F2 male (CpG2)  F2 male (CpG3) | AD (14.32)  PFF (13.76)  PFM (13.83)  AFF (13.1)  AFM (17.81)  AD (17.04)  PFF (18.68)  PFM (17.03)  AFF (16.75)  AFM (19.23)  AD (16.32)  PFF (16.37)  PFM (16.67)  AFF (15.5)  AFM (19.15) | AD vs AFM (2.468)  PFF vs AFM (2.864)  PFM vs AFM (2.814)  AFF vs AFM (3.331)  AD vs AFM (1.549)  PFF vs AFM (0.389)  PFM vs AFM (1.556)  AFF vs AFM (1.754)  AD vs AFM (2.00)  PFF vs AFM (1.966)  PFM vs AFM (1.754)  AFF vs AFM (2.581) | N=8  N=7  N=8  N=8 |
| 3E | F3 male (CpG1)  F3 male (CpG2)  F3 male (CpG3) | AD (14.26)  PFF (14.2)  PFM (13.88)  AFF (15.37)  AFM (17.81)  AD (14.98)  PFF (15.05)  PFM (14.38)  AFF (14.93)  AFM (15.71)  AD (16.33)  PFF (15.4)  PFM (15.66)  AFF (16.12)  AFM (20.28) | AD vs AFM (2.694)  PFF vs AFM (2.737)  PFM vs AFM (2.814)  AFF vs AFM (1.909)  AD vs AFM (0.516)  PFF vs AFM (0.467)  PFM vs AFM (0.941)  AFF vs AFM (0.552)  AD vs AFM (2.793)  PFF vs AFM (3.451)  PFM vs AFM (3.267)  AFF vs AFM (2.942) | N=8  N=8  N=8 |
